# Long-term ECMO, efficiency and performance of EUROSETS adult A.L.ONE ECMO oxygenator

**DOI:** 10.1186/s13019-023-02190-9

**Published:** 2023-03-30

**Authors:** Ignazio Condello, Roberto Lorusso, Giuseppe Nasso, Giuseppe Speziale

**Affiliations:** 1 Department of Cardiac Surgery, Anthea Hospital, GVM Care & Research, Via Camillo Rosalba 35/37, 70124 Bari, Italy; 2 Cardio-Thoracic Surgery Department, Heart and Vascular Centre, Maastricht University Medical Centre, Maastricht, The Netherlands; 3 Cardiovascular Research Institute Maastricht, Maastricht, The Netherlands

**Keywords:** Polymethylpentene, Hollow fibers, Extracorporeal membrane oxygenator, Long term, Efficiency, Performance, Safety

## Abstract

**Background:**

The management of the oxygenator can be prolonged in the long-term procedures especially during extracorporeal membrane oxygenation (ECMO) for bridge to transplant or bridge to recovery. Long-term use often involves an overrun of the time of use with respect to certification of the oxygenating module of 14 days, for the maintenance of performance and efficiency of the oxygenator. The evaluation of the long-term oxygenator efficiency is complex and depends on the: patient pathology, ECMO configuration, the management of coagulation and anticoagulation, materials selection and circuit components, the structure, design and performance of the oxygenator. In this context we investgated the long-term performance of the A.L.ONE Eurosets ECMO oxygenator in relation to the parameters prodromal to replacement.

**Methods:**

We retrospectively collected eight years data from Anthea Hospital GVM Care & Research, Bari, Italy on the long-term use exceeding 14 days of Eurosets A.L.ONE ECMO Adult oxygenator in Polymetylpentene fiber, for ECMO procedures, including the procedures: Veno Arterial (VA) ECMO post-cardiotomy or not, veno-venous (VV) ECMO. The primary end points were the evaluation of Gas Transfer: oxygen partial pressure (PO_2_) post oxygenator, Carbon dioxide partial pressure (PCO_2_) post oxygenator, the oxygen transfer across the oxygenator membrane V′O_2_, differential CO_2_ content across oxygenator; Pressure monitoring: oxygenator pressure Drop in relation to Blood flow rate (BFR) (ΔP); Hematologic values: Hemoglobin, Fibrinogen, Platelets, aPTT, D-Dimer, LDH.

**Results:**

Nine VA ECMO patients who used the oxygenator for 18.5 days and two VV ECMO patients who used the oxygenators for 17.2 days on the seventeenth days reported average values PaO_2_ (267 ± 29 mmHg); PaCO_2_ (34 ± 4 mmHg) with gas blender values set to 3.8 ± 0.6 L/min of air and a FiO_2_ of 78 ± 5%; the transfer across the oxygenator membrane V′O_2_ was 189 ± 43 (ml/min/m^2^). The mean peak of partial pressure of carbon dioxide from the gas exhaust of oxygenator (P_E_CO_2_) was 38 ± 4 mmHg; differential CO_2_ across the oxygenator “pre-oxygenator PCO_2_–post-oxygenator PCO_2_” (18 ± 6 mmHg); the mean blood flow rate (BFR) 4.5 ± 0.6 (L/minute); the pump revolution per minutes mean maximum rate was 4254 ± 345 (RPM); the mean pressure drop (ΔP) was 76 ± 12 mmHg; the mean peak of d-dimers (DDs) was 23.6 ± 0.8 mg / dL; the mean peak of LDH was 230 ± 55 (mg/dl); fibrinogen mean peak 223 ± 40 (mg/dl).

**Conclusions:**

The performance of the Eurosets A.L.ONE ECMO Adult polymethylpentene fiber oxygenator in our experience has proven efficiency in terms of O_2_ uptake and CO_2_ removal, blood fluid dynamics, metabolic compensation and heat exchange in the long-term treatment. The device was safe without iatrogenic problems over a period of 14 days in the patients undergoing ECMO VA and in all patients undergoing VV ECMO with continuous administration of anticoagulation therapy.

## Introduction

With improvements in circuit technology and expanding supportive evidence, extracorporeal membrane oxygenation (ECMO) use has grown rapidly over the past decade. The management of the oxygenator can be prolonged in the long-term procedures especially during extracorporeal membrane oxygenation (ECMO) for bridge to transplant or bridge to recovery. In this study, we present a classification of the short, medium, long term use of the oxygenating module in relation to its certification and validation [1]. Long-term use often involves an overrun of the time use respect to certification with only one oxygenating module. The evaluation of the long-term oxygenator efficiency is complex and depends on the type of: patient pathology, ECMO configuration, the management of coagulation or anticoagulation, materials selection; circuit components and design, the structure, design and performance of the oxygenator [2]. In this study we present a retrospective analysis about Eurosets A.L.ONE ECMO Adult Polymethylpentene fiber oxygenator a medical device validated and certified by the manufacturer (Eurosets SPA, Medolla, Italy) for ECMO procedures up to 14 days. In this context we wanted to investigate the long-term performance of the A.L.ONE Eurosets ECMO oxygenator in relation to the parameters prodromal to replacement: Hematologic profiles (Coagulopathy, Hemolysis) [1–3]; Pressure monitoring (Blood Flow, Pressure Drop); Gas Transfer (O_2_ uptake and CO_2_ removal) [4, 5].

## Materials and methods

### Extracorporeal membrane oxygenation settings

**Fig. 1:**
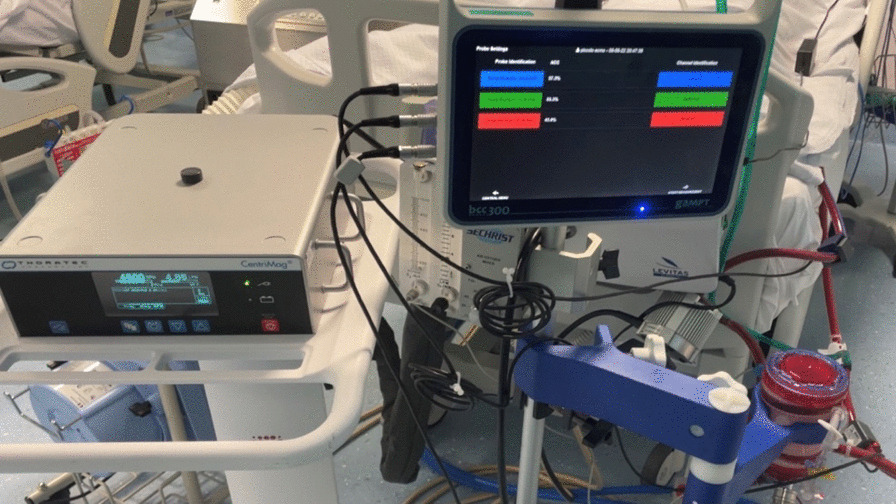
Adult A.L.ONE ECMO Oxygenator (Eurosets SPA, Medolla, Italy) configuration with Thoratec Centrimag, centrifugal magnetic levitation pumps (Abbott) during VA ECMO

**Fig. 2:**
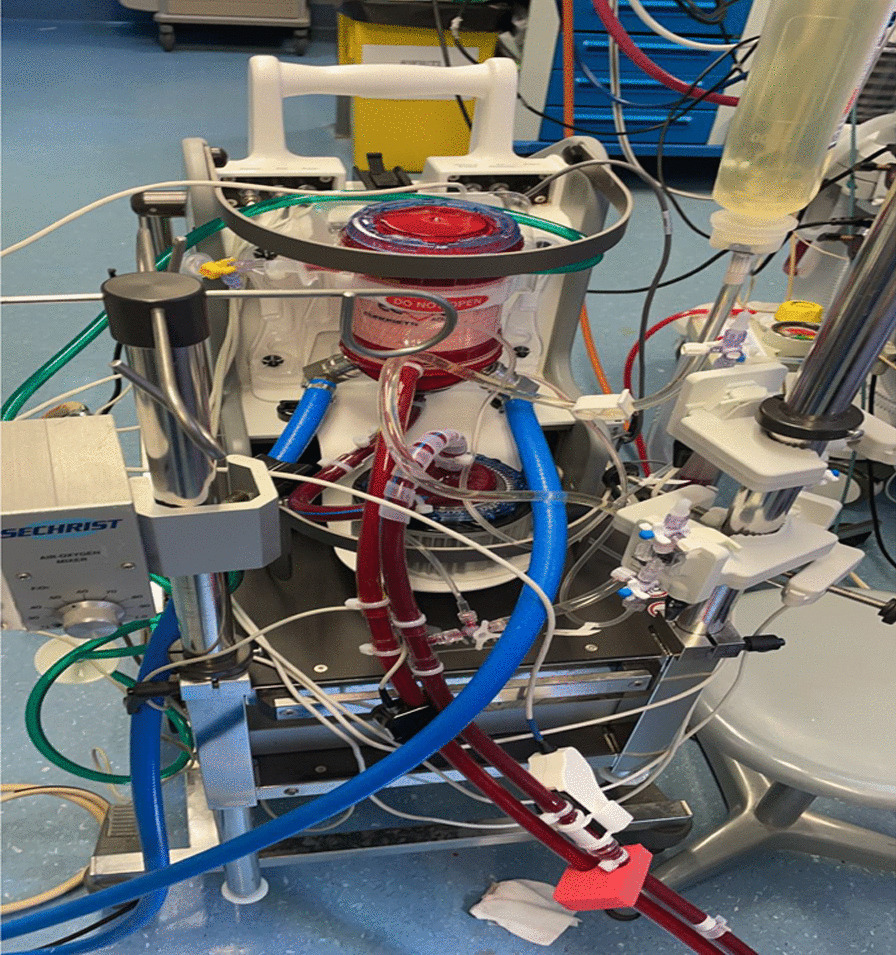
Adult A.L.ONE ECMO Oxygenator configuration with ECMOLIFE, centrifugal magnetic levitation pump (Eurosets SPA, Medolla, Italy) during VA ECMO

**Fig. 3:**
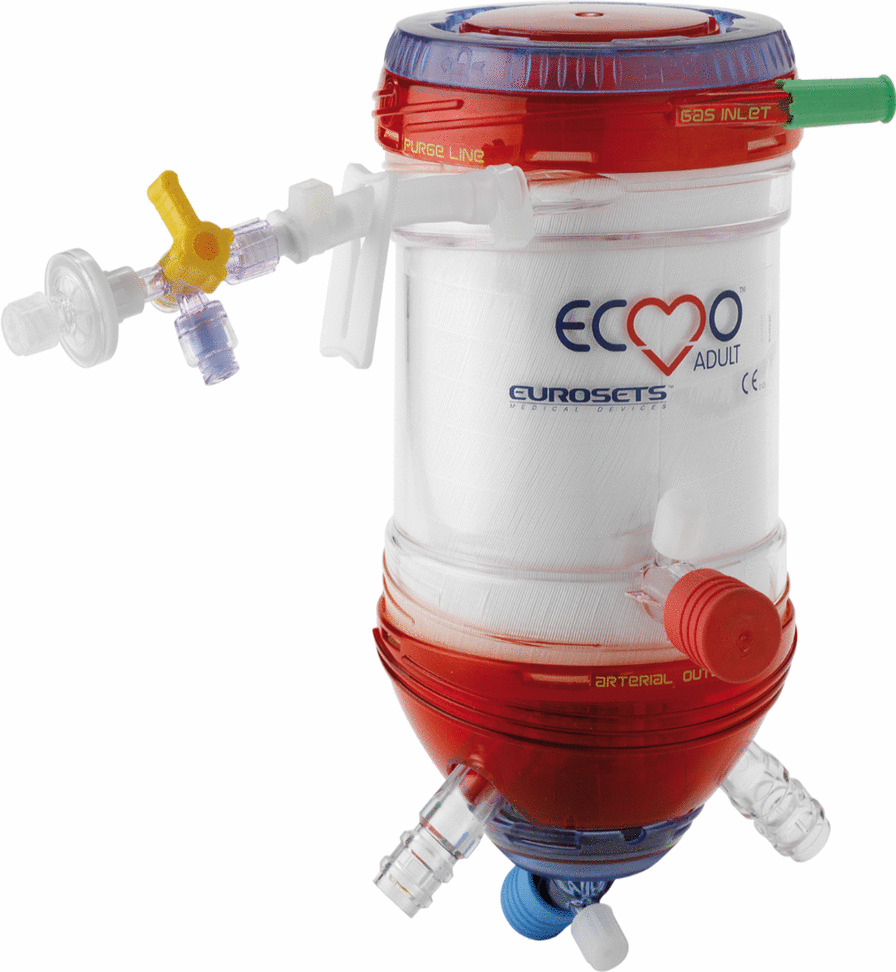
Adult A.L.ONE ECMO Oxygenator (Eurosets SPA, Medolla, Italy)

**Fig. 4:**
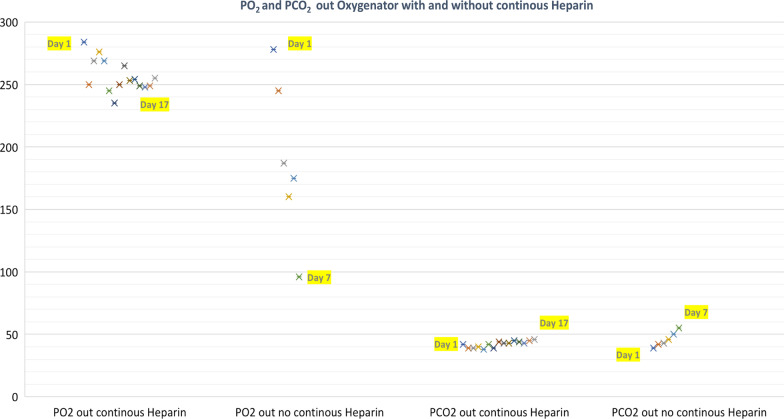
Trend of PO_2_ and PCO_2_ out Oxygenator with and without continuous Heparin

The ECMO circuit consists exclusively of commercially available components. By default, a Thoratec Centrimag centrifugal magnetic levitation pumps (Abbott) (Fig. 1) and ECMOLIFE centrifugal magnetic levitation pumps (Eurosets SPA, Medolla, Italy) (Fig. 2) were used, in synergy with Landing system for pressure monitoring (Blood Flow, Pressure Drop); Gas Transfer (O_2_ uptake and CO_2_ removal), (Eurosets SPA, Medolla, Italy). As a standard, the Eurosets A.L.ONE ECMO Adult oxygenator was used (Fig. 3). The tubing and the oxygenator were treated with phosphorylcholine-coated surface (Eurosets SPA, Medolla, Italy). The system has a priming volume of 500 ml and features connectors for other emergency extracorporeal devices, such as renal replacement devices or rapid infusion systems for advanced in-center intensive care treatment during further courses of therapy. The main determinants of cannula sizing in peripheral VA ECMO are anatomical considerations and the targeted flow rate. Generally, cannulas are chosen to support a flow equivalent to a cardiac index of 2.2–2.5 L/m^2^/min, which is considered full flow. Femoral Arterial cannulas that we used were 17–25 Fr and Femoral venous cannulas were usually 19–25 Fr Biomedicus (Medtronic, Minneapolis, USA). For Central VA ECMO Aortic Arterial cannulas were 20–24 Fr EOPA (Medtronic, Minneapolis, USA) and Atrial venous cannulas were 32/40–36/46 Fr (Medtronic, Minneapolis, USA). For Peripheral VV ECMO for Femoral venous cannulas were 19–25 Fr for reinfusion in jugular vein cannulas were 17–21 Fr Biomedicus (Medtronic, Minneapolis, USA) (Fig. 4).

### Anticoagulation and blood product management

The ECMO patients are i our center are anticoagulated with a heparin infusion, with a goal activated partial thromboplastic time (aPTT) of 50–65 s unless the clinical setting (e.g., active bleeding) dictates otherwise. The heparin infusion is titrated with a nurse-managed nomogram, whereby the initial infusion dose is based on the patient’s weight. Six hours after the infusion begins, an aPTT is again drawn, and the rate of infusion is increased if subtherapeutic (< 50 s), decreased if supratherapeutic (> 65 s), or kept constant if within goal (50–65 s). Another aPTT is drawn in 6 h until the second consecutive aPTT is within target range, at which point the aPTT is checked daily. In our institution the usual practice is to transfuse platelets when counts fall below 80,000/μL, although several experienced centers use a more conservative approach and transfuse platelets only when they fall below 40,000–50,000/μL, or even as low as 20,000 in non- bleeding patients. The strategies in RBC transfusion depending on Hb level—restrictive when transfusion is performed at a Hb level of 7–9 g/dL, and liberal with a Hb level between 10 and 12 g/dl, in relation to Blood Flow (BF), Cardiac Output (CO) and Oxygen Delivery (DO_2_) [6].

### Veno-arterial (VA) ECMO indication

ECMO was initiated for circulatory instability during or immediately after weaning from the cardiopulmonary bypass (CPB) in the primary cardiac procedure or for hemodynamic support for high risk interventional cardiology procedures. The clinical criteria for hemodynamic support included the following: left atrial pressure > 15 mmHg; central venous pressure > 12 mmHg; metabolic acidosis (i.e. pH < 7.3 with serum lactate > 3.0 mmol/L); end-organ hypoperfusion (urine output < 30 mL/h); cardiac index < 2.2 L/min/m^2^; and systolic blood pressure < 80 mmHg despite adequate filling volumes, use of multiple adrenergic agents (epinephrine > 0.1 µg/kg/min or dobutamine > 10 µg/kg/min, norepinephrine > 0.1 µg/kg/min), or an intra-aortic balloon pump (IABP). VA-ECMO support was initiated via peripheral cannulation through the femoral route with the semi-open method, and an additional 6 Fr catheter was systematically inserted distally into the femoral artery to prevent leg ischemia ECMO blood flow was adjusted on based on clinical assessments (e.g. pre-oxygenator venous oxygen saturation, evidence of hypoperfusion, resolution of hyperlactatemia, normalization of mean arterial pressure). ECMO-related complications were carefully monitored. ECMO weaning was performed in patients who fulfilled our published institutional weaning criteria and passed an ECMO weaning trial consisting in decreasing and clamping ECMO flow. In general, the patient should have a pulsatile arterial waveform for at least 24 h; be hemodynamically stable, with baseline mean arterial pressure greater than 60 mmHg with no or low doses of catecholamines; should have left ventricular ejection fraction (LVEF) of 35%, and an aortic velocity time integral (VTI) of ≥ 12 cm; and have recovered from major metabolic disturbances. Weaning was considered unsuccessful if ECMO re-cannulation was required within 2 days of decannulation [2–7].

### Veno-venous (VV) ECMO indication

The indication for VV-ECMO are typically severely hypoxemic and/or hypercapnic and unresponsive to optimal medical management, including protective ventilation with low-tidal volumes and plateau pressure less than 28–30 cmH_2_O, high levels of PEEP, prone positioning, neuromuscular blockers and/or other adjunctive therapies, including nitrous oxide or almitrine. The recent literature suggests that a PaO_2_/FIO_2_ ratio of 70–80 mmHg, Murray score > 3, and pH < 7.2 provide a reasonable threshold for considering VV-ECMO in adults with ARDS. It is crucial to determine the acute nature of the pulmonary failure, exclude cardiac and/or other organ failure and verify that the respiratory failure cannot be improved with optimal ventilator management [8].

### Indication and cut-off parameters used for oxygenator or circuit replacement

The polymethylpentene fiber oxygenator is responsible for oxygen uptake and carbon dioxide removal. The non-biologic surface of the oxygenator activates inflammatory and coagulation pathways with thrombus formation, fibrinolysis, and leukocyte activation leading to fiber dysfunction. Activation of coagulation and fibrinolysis can precipitate systemic coagulopathy or hemolysis, while clot deposition can obstruct blood flow. Additionally, moisture buildup in the gas phase and protein and cellular debris accumulation in the blood phase may contribute to shunt and dead-space physiology, respectively, impairing gas exchange. These three categories—hematologic abnormalities, mechanical obstruction, and inadequate gas exchange—prompt the majority of oxygenator exchanges. Principal Cut-off parameters for replacement the oxygenator or the circuit, Gas Transfer: Arterial oxygen partial pressure (PO_2_) post oxygenator (< 200 mmHg), Carbon dioxide partial pressure PCO_2_ (> 40 mmHg) post oxygenator, the oxygen transfer across the oxygenator membrane V′O_2_ (< 100–150 ml/min/m^2^),

$$V^{\prime } O_{2} = BFR\left( {CPostO_{2} - CPreO_{2} } \right)$$

where V′O_2_ = O_2_ transfer across the oxygenator (mL/min), BFR = blood flow rate (L/min), Cx O_2_ = O_2_ content of(pre-/post-oxygenator) blood (mL/L) for

$$CxO_{2} = 13.4 \cdot Hb \cdot SxO_{2} + 0.03 \cdot PxO_{2}$$

where Hb = hemoglobin (g/dL), Sx O_2_ = O_2_ saturation of (pre-/post-ML) blood, Px O_2_ = O_2_ partial pressure of (pre-/post-oxygenator) blood (mmHg).

Measurement of V′O_2_ provides an objective measure of oxygen transfer and can confirm oxygenator dysfunction, when clinically indicated. Differential CO_2_ across the oxygenator “pre oxygenator PCO_2_–post oxygenator PCO_2_” (< 10 mmHg); Pressure monitoring: pressure Drop across the oxygenator “Pressure Pre oxygenator–Pressure Post oxygenator” (> 80 mmHg) in relation to Blood flow rate (BFR) (ΔP); Hematologic profiles: Fibrinogen (< 200 mg/dl), Platelets (< 80,000 10^9^/L), aPTT (> 65 s), D-Dimer (> 25–30 ng/ml), LDH (> 250 mg/dl) [1].

### Patients and data collection

We recruited retrospectively from January 2014 to May 2022 at Institution of Anthea Hospital GVM Care & Research, Bari, Italy, long-term ECMO procedures (exceeding 14 days) that use the Eurosets A.L.ONE ECMO Adult oxygenator. The procedures analyzed including: Veno-arterial (VA) ECMO post-cardiotomy or not, veno-venous (VV) ECMO. ECMO characteristics are described and presented as means with sd or medians with interquartile range. The primary end point was the substitution of oxygenator incidence in relation to the oxygenator performance were Gas Transfer: O_2_ uptake and CO_2_ removal were collected in relation to the blood flow rate (BFR), maximum rate per minute of pump (RPM), hemoglobin value (Hb), ventilation indices FiO_2_ (%)/ Air (L/min), PO_2_ post oxygenator (mmHg), PCO_2_ post oxygenator, the transfer across the oxygenator membrane V′O_2_ (ml/min/m_2_), Indexed Oxygen Delivery (_i_DO_2_) (ml/min/m^2^) only for VA ECMO patient, the partial pressure of carbon dioxide from the gas exhaust of oxygenator (P_E_CO_2_) (mmHg), differential CO_2_ across the oxygenator (mmHg); Hematologic profiles: Fibrinogen (mg/dl), Platelets (10^9^/L), aPTT (sec), D-Dimer (ng/ml), LDH (mg/dl), and incidence of Heparin‐induced thrombocytopenia (HIT) I, II, Temperature in arterial and venous line (°C) and Pressure monitoring: pressure Drop (ΔP) (mmHg).

### Statistical analysis

Continuous data were expressed as mean ± standard deviation or a median with the interquartile range and categorical data as percentages. Cumulative survival was evaluated with the Kaplan–Meier method. All reported *p*-values were two-sided, and *p*-values of < 0.05 were considered to indicate statistical significance. All statistical analyses were performed with SPSS 22.0 (SPSS, Inc., Chicago, IL, USA).

## Results

From January 2014 to May 2022 twenty-two ECMO procedures with Eurosets A.L.ONE ECMO Adult Polymethylpentene fiber oxygenator were retrospectively collected from the tertiary institution Anthea Hospital GVM Care & Research, Bari, Italy. Eight peripheral VA ECMOs were used as short-term hemodynamic support for interventional cardiology procedures, the medium time of use was less than 3 h. Twelve VA ECMOs were used as cardiocirculatory assistance, nine post cardiotomy of which: two with central cannulation, seven with peripheral cannulation and one peripheral after angioplasty. In nine oxygenators with continuous endo venous heparin treatment the average use time was 18.5 days. Only three oxygenators on post-cardiotomy VA ECMO procedures without continuous endo venous heparin treatment for patient bleeding were replaced on three patients between the sixth/seventh day of use for the performance decrease for previously mentioned cut-off (Table 1). in particular, the failure of the oxygenator was mainly caused by oxygenation (PO_2_ out the oxygenator < 120 mmHg with 100% FIO_2_ set to 10 L of gas flow), decapneization (PCO_2_ out the oxygenator > 45 mmHg with 10 L of gas flow and 100% FiO_2_) (Fig. 3), by the pressure drop (> 350 mmHg), by the reduction of the pump flow rate (< 2.4 l/min/m2) and by the visible formation of clots in the part corresponding to the purging of the oxygenator because an area with low vorticity which could have facilitated the halving of the platelet count (< 100,000 10^9^/L). None of the patients reported incidence of HIT I and II. The use of a Phosphorylcholine-treated ECMO circuit versus a heparin circuit likely reduces heparin exposure by reducing the incidence of HIT [9]. Two oxygenators were used on Veno-Venous (VV) ECMO with continuous endo venous heparin treatment for the treatment of Acute Respiratory Distress Syndrome (ARDS) the average use time was 17.2 days. The 9 VA ECMO patients who used the oxygenator for 18.5 days and the two VV ECMO patients who used the oxygenators for 17.2 days on the seventeenth days reported average values of mean hemoglobin 8.9 ± 0.8; PO_2_ post oxygenator (267 ± 29 mmHg); PCO_2_ post oxygenator (34 ± 4 mmHg) with average values of gas blender set to Air 3.8 ± 0.6 L/min and a FiO_2_ of 78 ± 5%; the oxygen transfer across the oxygenator membrane V′O_2_ was 189 ± 43 (ml/min/m^2^); the mean peak of indexed oxygen delivery (_i_DO_2_) for only nine VA ECMO at seventeenth days was 340 ± 37 ml/min/m^2^. The mean peak of partial pressure of carbon dioxide from the gas exhaust of oxygenator (P_E_CO_2_) was 20 ± 4 mmHg; differential CO_2_ across the oxygenator “pre-oxygenator PCO_2_–post-oxygenator PCO_2_” was 18 ± 6 (mmHg); the mean blood flow rate (BFR) was 4.5 ± 0.6 (L/minute); the pump revolution per minutes mean maximum rate was 4254 ± 345 (RPM); the mean pressure drop (ΔP) was 76 ± 12 mmHg; the mean arterial blood temperature was 36.5 ± 0.3 °C and in the venous line was 36.4 ± 0.2 °C; the mean peak of D-dimers (DDs) was 23.6 ± 0.8 mg/dL; the mean peak of LDH was 230 ± 55 (mg/dl); fibrinogen mean peak 223 ± 40 (mg/dl); PLT 150,000 ± 987 (10^9^/L); aPTT 53 ± 4 (sec), the other hematologic values data prodromal to oxygenator replacement are presented in the Table 1.

**Table 1:** Operative data, during long term versus medium term on Adult A.L.ONE ECMO Oxygenator

Procedures nr = 14	Long term VA (Nr = 9) and VV ECMO (Nr = 2)	Medium term VA ECMO(Nr = 3)	*p-value*
	Parameters at seventeenth day	Parameters at sixth day	
Period (days)	17.8	6.8	0.003
Hemoglobin (gr/dl)	8.9 ± 0.8	7.3 ± 0.8	0.022
PO_2_ post oxygenator (mmHg)	267 ± 29	170 ± 23	0.002
PCO_2_ post oxygenator (mmHg)	34 ± 4	42 ± 8	0.034
Air (L/min) /FiO_2_ (%)	3.8 ± 0.6/78 ± 5	5.3 ± 0.9/100 ± 9	0.019
V′O2 (ml/min/m^2^)	189 ± 43	105 ± 28	0.017
_i_DO_2_ (ml/min/m^2^)	340 ± 37	245 ± 48	0.025
P_E_CO_2 (mmHg)_	20 ± 4	10 ± 6	0.031
Differential CO_2_ (mmHg)	18 ± 6	8 ± 3	0.021
BFR (L/min)	4.5 ± 0.6	3.9 ± 0.9	0.041
Pump revolution (RPM)	4254 ± 345	5000 ± 445	0.023
Pressure drop (ΔP)	76 ± 12	246 ± 22	0.011
Arterial blood temperature (°C)	36.5 ± 0.3	36.2 ± 0.8	0.89
Venous blood temperature (°C)	36.4 ± 0.2	36.1 ± 0.5	0.91
Continuous anticoagulation Use	Yes	No	
DDs (mg/dL)	23.6 ± 0.8	42 ± 13	0.005
LDH (mg/dL)	230 ± 55	424 ± 38	0.004
Fibrinogen (mg/dL)	223 ± 40	176 ± 40	0.003
PLT (10^9^/L)	150,000 ± 987	39,000 ± 778	0.033
aPTT (sec)	53 ± 4	69 ± 23	0.018
Oxygenator replacement (nr)	0	3	0.002
Total oxygenator used (nr)	11	6	0.033
Red blood cells (units for patients)	2 ± 1	2 ± 1	0.027
Platelets (units for patients)	3 ± 1	2 ± 1	0.027

Values are presented as n (%) or mean ± standard deviation

*VA*, Veno-Arterial; *VV*, Veno-venous; *ECMO*, extracorporeal membrane oxygenation; *PO*_*2*_, partial pressure of oxygen; *PCO*_*2*_, partial pressure of carbon dioxide; *V′O*_*2*_, the oxygen transfer across the oxygenator membrane; _*i*_*DO*_*2*_, indexed oxygen delivery; *P*_*E*_*CO*_*2*_, partial pressure of carbon dioxide from the gas exhaust of oxygenator; *BFR*, blood flow rate; *DDs*, D-Dimers; *LDH*, lactate dehydrogenase; *PLT*, Platelets; *aPTT*, partial thromboplastin time

## Discussion

The main limitation of this study is the fact that it is a single center retrospective investigation with a small number of ECMO cases. An advantage of a small sample size in this case may be to contain medical, technical, and nursing management skills compared to a multicenter study. Long-term oxygenator management during post-cardiotomy ECMO procedures in the literature is limited with a high incidence of replacement due in particular to post-procedures bleeding [10, 11]. In future perspective would be interesting to explore this issue further in a larger multicenter study.

In this study, we present a classification of the short, medium, long term use of the oxygenating module in relation to its certification and validation and demonstrate that the determinant that can impact the duration of the oxygenator and its failure in particular after cardiac surgery procedures does not depend exclusively on the model and design but mainly on the medical and technical management of the device in relation to the anticoagulant. The same model in this case it may have a different duration and need to be replaced depending on the suspension or continued use of heparin infusion [9]. Long-term use often involves an overrun of the time use respect to certification with only one oxygenating module. The evaluation of the long-term oxygenator efficiency is complex and depends on the type of: patient pathology, ECMO configuration, the management of coagulation or anticoagulation, materials selection; circuit components and design, the structure, design and performance of the oxygenator. Prompt recognition of oxygenator dysfunction is vital for safety, allowing for elective replacement in a controlled manner [10, 12]. On the other hand, replacement of an adequately functioning device requiring temporary cessation of ECMO support places the patient at unnecessary risk while consuming a limited and expensive resource. Based on the pathophysiology of the oxygenator, replacement may be required for one of three reasons: if there is (A) an associated hematologic abnormality, (B) an increasing obstruction to blood flow, or (C) inadequate gas exchange [1]. In our experience we report that the continuous anticoagulation and a good balance in aPTT management is protective for the oxygenators until long-term use respect to the group that suspend the anticoagulation strategy for bleeding.

## Conclusions

The performance of the Eurosets A.L.ONE ECMO Adult polymethylpentene fiber oxygenator in our experience has proven efficiency in terms of O_2_ uptake and CO_2_ removal, blood fluid dynamics, metabolic compensation and heat exchange in the long-term treatment. The device was safe without iatrogenic problems over a period of 14 days in the patients undergoing ECMO VA and in all patients undergoing VV ECMO with continuous administration of anticoagulation therapy, oxygenator replacement was not reported in this group of patients, compared with three replacements in the group that did not do continuous heparin infusion for bleeding.

## Data Availability

The datasets analyzed during the current study are available from the corresponding author on reasonable request.
